# Mechanical properties of steel slag replaced mineral aggregate for road base/sub-base application based Vietnam and Japan standard

**DOI:** 10.1007/s11356-021-16706-0

**Published:** 2021-12-02

**Authors:** Dang Tung Dang, Manh Tuan Nguyen, Tan Phong Nguyen, Tomoo Isawa, Yasutaka Ta, Ryoichi Sato

**Affiliations:** 1 Department of Bridge and Highway, Faculty of Civil Engineering, Ho Chi Minh City University of Technology (HCMUT), Ho Chi Minh City, Vietnam; 2 Vietnam National University Ho Chi Minh City, Ho Chi Minh City, Vietnam; 3 Faculty of Environment-Natural Resources and Climate Change, Ho Chi Minh City University of Food Industry, Ho Chi Minh City, Vietnam; 4 Steel Research Lab, JFE Steel Corporation, Kawasaki, Japan

**Keywords:** Steelmaking slag, Industrial by-product, Slag recycling, CBR test, Elastic modulus test

## Abstract

Steelmaking slag is one of the most massive industrial by-products generated during steelmaking processes. This paper presents the current steelmaking slag production status and its potential to use as mineral aggregates in base/sub-base layer of road pavement. The mechanical properties of steelmaking slag were confirmed by the test method specified in Vietnam specification. The volume stability test of the slag was conducted based on JIS A 5015-2018 (Japanese Industrial Standard: Iron and steel slag for road construction). From the results, it was confirmed that steelmaking slag can satisfy all the mechanical requirements specified in Vietnam specification and the requirements regarding stability specified in JIS A 5015-2018. In addition, it was found that the elastic modulus of steelmaking slag applied as a base or sub-base layer in pavement was higher than that of the conventional graded aggregate made from mineral aggregate. Therefore, the thickness of pavement can be reduced by using steelmaking slag, and the construction cost can be lower.

## Introduction

Application of industrial waste or by-product in highway construction is a major topic by researchers, government officers, and engineers these days (Dang et al. 2021). Steelmaking slag is a by-product of metallurgical processing and is typically generated from processing of steel. It can be categorized as carbon steel slag and stainless steel slag according to the type of steel, and as pre-treatment slag, basic oxygen furnace slag, electrical arc furnace slag, induction furnace slag, ladle refining slag, and casting residue according to the steelmaking process (Huang et al. 2012). Approximately 150–200 kg steelmaking slag is produced when manufacturing one ton of crude steel. According to Nguyen (2016), the steel production was about 12.6 million tons per year in Vietnam. As a result, the steelmaking slag output a year is over 2 million tons, and they could cost over 20 million US dollar for handling. On the other hand, the good quality of aggregate sources from natural rock mountain in Ho Chi Minh City or Vietnam decreases these days due to their applications in constructions. Then, application of slags as the alternative material of mineral aggregate is necessary for sustainable development in Vietnam.

Huang et al. (2012) showed that the steel and steel slag annual output of 2010 in China reached to 626.7 million tons and 90 million tons respectively and the utilization rate of steel slag in China is about 22%, far behind the developed countries like the USA, Japan, Germany, and France, of which the rates have been close to 100%. In these developed countries, about 50% of slag has been used for the road project directly, and another part for sintering and iron-making recycling in iron and steel manufacturing plant.

In the EU and North America, steel slag is used in many field of road structures such as bitumen bound materials, pipe bedding, hydraulically bound mixtures for sub-base and base, unbound mixtures for sub-base, capping, embankments and fill construction, clinker manufacture and fertilizer and soil improvement agent (Haritonovs et al. 2012). In the USA, the sales of ferrous slags in 2005 (Meg 2019) were collected and applied in road bases and surfaces which have 34% of air-cooled blast furnace slag and 53% of steelmaking slag. In Japan, iron and steel slag is now used in road construction. Through JIS A 5015, iron and steel slag can be used for road bed and for heated asphalt mixture. Haritonovs et al. (2010) and Haritonovs and Tihonovs (2014) proved that steel slag aggregate in asphalt mixtures can improve the rutting performance.

In Vietnam, many researchers focus on using the steel slag as cementitious materials (Nguyen et al. 2014), and replace natural aggregate in geopolymer concrete (Dong and Son 2019), as well as asphalt concrete (Nguyen and Le 2018). The combinations of steel slag and natural aggregate even showed better performance than asphalt concrete made entirely of steel slags (Nguyen and Le 2018), but the application or studies of steel slag into base/sub-base layer have not been concerned properly.

As the result, this paper tried to use steelmaking slag from JFE Steel Corporation for replacing mineral aggregates in base/sub-base layer of road pavement based on Vietnam specifications. The important mechanical properties of steel slag, which are Los Angeles abrasion, and CBR test, were conducted. The immersion expansion of the slag was conducted based on JIS A 5015 in order to prevent from expansion during in service because of lacking this method from Vietnam specification. Besides, the resilient modulus of steel slag also was checked in a small concrete hole as a pilot section.

## Experimental program and results

### Material

**Fig. 1:**
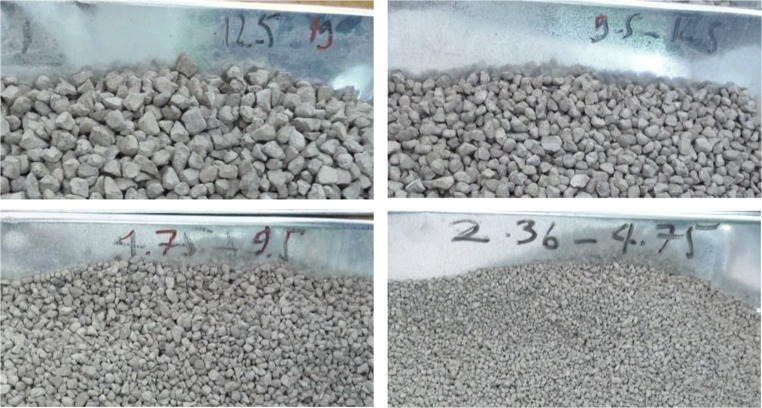
Steelmaking slag used in the study

In this study, the basic oxygen furnace slag imported from JFE Steel Corporation (Japan) was used as shown in Fig. 1. The Los Angeles (LA) abrasion test was carried to obtain an indication of the desired toughness and abrasion characteristics of steel slag based on TCVN 7572-12 (2006) (or AASHTO T96). Three gradation types including B, C, D which weights are shown in Table 1 were chosen to check the LA abrasion test. After being subjected to the rotating drum for 500 cycles, the weight of aggregate that passed on a No.12 (1.70 mm) sieve was the abrasion aggregate. Besides, a good natural aggregate source in Tan Dong Hiep, Binh Duong province, Vietnam, was conducted for the LA test and the LA value was 18.9% for B type. The LA test value from natural aggregate was bigger than steelmaking slag LA value, shows that the steelmaking slag was better than natural aggregate in terms of abrasion loss test. The chosen slag gradation was used in this study shown in Table 2 based on TCVN 8857. This gradation was used for the entire following test after this part.

**Table 1:** Weight of sample for LA abrasion test and test result

Sieve size (mm)		Weight (g)		
		B type	C type	D type
37.5–25		-	-	-
< 25–19		-	-	-
< 19–12.5		2500 ±10	-	-
< 12.5–9.5		2500 ±10	2500 ±10	-
< 9.5–6.3		-	2500 ±10	-
< 6.3–4.75		-	-	2500 ±10
< 4.75–2.36		-	-	2500 ±10
Total		5000 ±10	5000 ±10	5000 ±10
LA abrasion loss (%)	Steelmaking slag	13.9	15.0	17.2
	Natural aggregate	18.9	-	-

**Table 2:** Slag gradation

Gradation type	Percent passing (%)					
	25 mm (1 in.)	9.5 mm (3/8 in.)	4.75 mm (No.4)	2.0 mm (No.10)	0.425 mm (No.40)	0.075 mm (No.200)
Limit from TCVN 8857	100	50–85	35–65	25–50	15–30	5–15
Study gradation	100	50	35	25	15	5

### Mix design

The optimal moisture content of steel slag was conducted based on 22TCN 333 (2006) (or AASHTO T180). This test method determines the relationship between the moisture content and the dried density of slag compacted in a mould by Proctor device. According to the result of gradation in Table 2, method II-D or modified proctor method was chosen in this study. The specimen mould has 152.4 mm in diameter. In order making a specimen, Hobart mixer was used to mix the gradation of steel slag and water together. Five water contents were chosen from 6 to 10% and five dried densities were determined. From relationship between moistures and dried densities, the optimal content and dried density were 8.3% and 2.63 g/cm^3^, respectively.^.^

### California Bearing Ratio

**Fig. 2:**
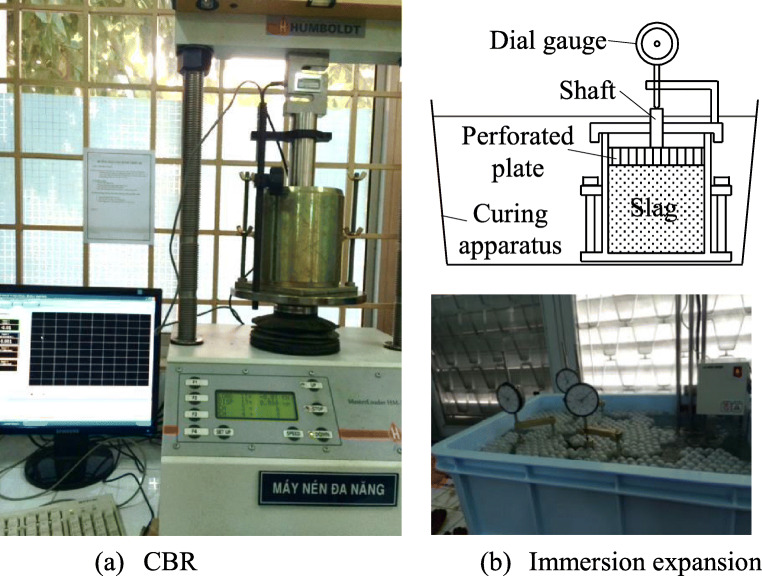
CBR and immersion expansion test set-up. **a** CBR. **b** Immersion expansion

The California Bearing Ratio (CBR) test was conducted based on Vietnamese standard for CBR 22TCN 332-06 (2006) (or AASHTO T193). Three specimens were compacted with 5 layers at 10, 30, and 65 blows of each layer. Before doing CBR test, three specimens were put in the water tank for 4 days. The loading machine was equipped with a movable head or base that travels at a uniform rate of 1.27 mm/min for use in forcing the penetration piston into the specimen as shown in Fig. 2(a). Based on relation between pressure and penetration, the CBR value at 0.98 of compaction degree was 50.80%.

### Immersion expansion test

According to JIS A 5015, the immersion expansion ratio shall be calculated by the following formula:

1 $$ {\gamma}_e=\frac{D_F-{D}_S}{H}100 $$

where *γ*_e_ = immersion expansion ratio (%); *D*_F_ = the last reading of the dial gauge (mm);

*D*_S_ = the first reading of the dial gauge (mm);*H* = the first height of the specimen (mm).

The test was carried out on three specimens prepared from the sample taken at the same time. In this study, we used the CBR mould with 116.43 mm of height to perform the expansion test instead of 125 mm in height based on JIS A 5015. The immersion expansion test first was conducted at 80 ± 3°C for 6 h, then leave it to cool in the curing apparatus. Then, the operation was repeated at 80 ± 3°C for 6 h, and one time per day for 10 days as shown in Fig. 2(b). The immersion expansion test result in Table 3 shows that the value *γ*_e_ was less than 1.0% and met the value specified in JIS A 5015-2018.

**Table 3:** Immersion expansion test result

Replicate	*D*_F_ (mm)	*D*_S_ (mm)	*H* (mm)	Immersion expansion ratio ***γ***_e_ (%)
1	3232	3196	116.43	0.31
2	3131	3056	116.43	0.64
3	3821	3753	116.43	0.58

### Resilient modulus

**Fig. 3:**
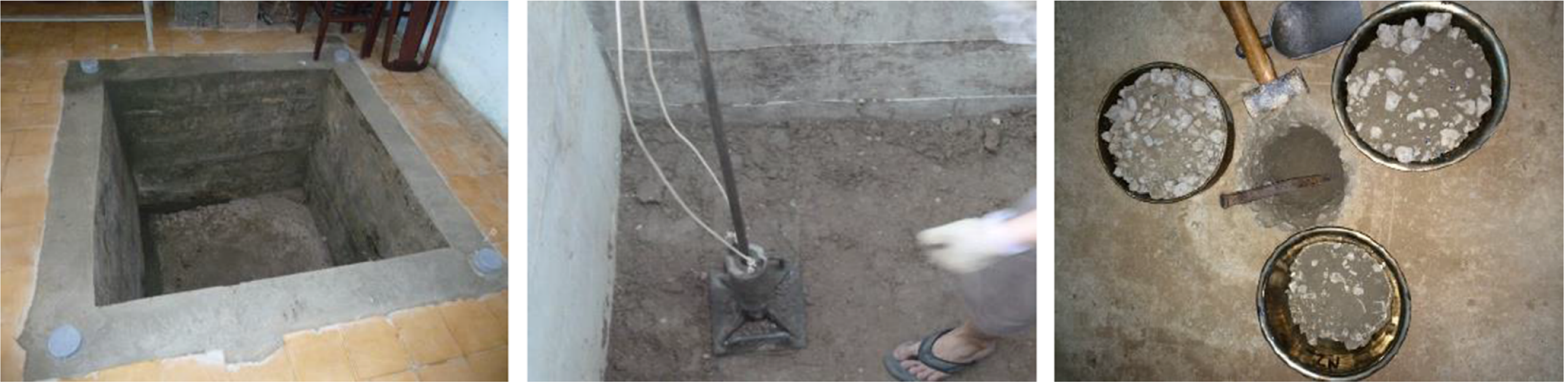
Reinforced concrete hole, hand compactor, and measure the degree of compaction

In order to having the resilient modulus of iron and steel slag used in sub-base layer in Vietnam, a small pilot section or small hole which sizes were about 1.64 m, 1.42 m, and 1.42 m in height, length, and width, respectively, was created. The hole was covered by reinforced concrete as shown in Fig. 3. After time curing of the reinforced concrete hole, the materials poured to the hole by hand compactor in Fig. 3 in order to simulate the road embankment.

The sand soil was in the bottom of the hole to simulate the subgrade. Over the sand soil, the natural soil with crush stone was used to simulate the capping layer that create a good bed road to easily compact other pavement structures above. After compaction, the degree of compaction of each layer was measured as shown in Fig. 3 to check the quality of compaction and the results show that the measured values are better than 98%.

**Fig. 4:**
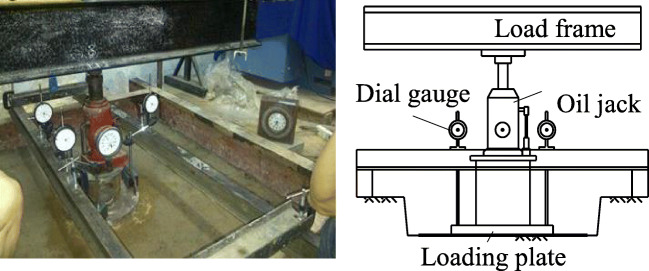
Resilient modulus test from concrete hole

**Fig. 5:**
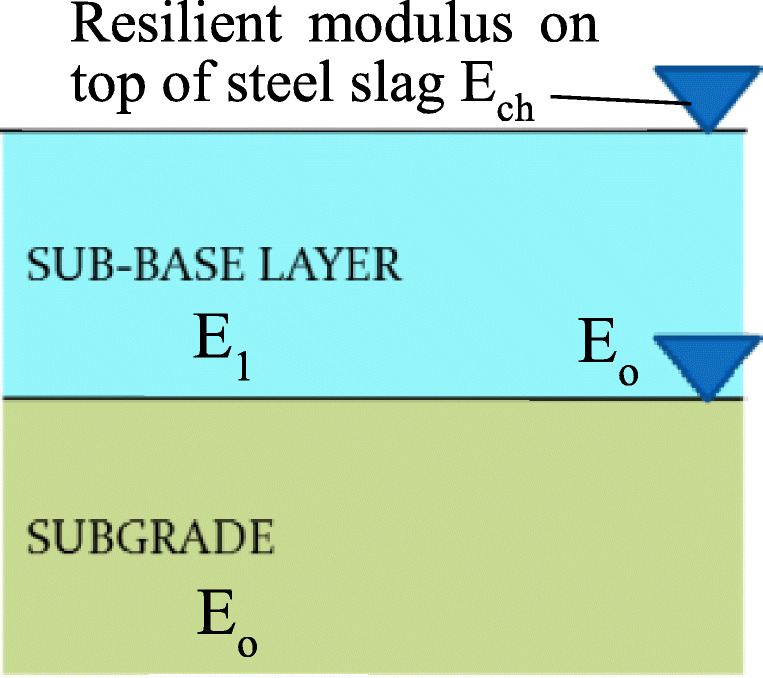
Relation of resilient modules

The set-up test for resilient modulus was conducted as shown in Fig. 4. The resilient modulus of subgrade (*E*_o_), which is shown in Fig. 5, was measured based on TCVN 8867 (2011) at 9 positions in concrete hole and average value was 276 MPa. After that, the steel slag was placed over the subgrade and resilient modulus on top of steel slag (*E*_ch_) was measured. The average value *E*_ch_ was 317 MPa. All test results which are the measured values and average values are presented in Table 4.

**Table 4:** Resilient modulus test result

Replicate	1	2	3	4	5	6	7	8	9	Ave.
*E*_o_ (MPa)	288	305	288	306	241	277	299	267	213	276
*E*_ch_ (MPa)	298	316	318	303	398	287	352	265	316	317
*E*_1_(MPa) with 95% of confidence level 268										

According to 22TCN 211 (2006) or relationship between resilient moduli based multi-layer elastic theory, the average resilient modulus of steel slag (*E*_1_) shall be calculated by the following formula:

2 $$ {E}_{ch}=\frac{\left[1.05-0.1\frac{h}{D}\left(1-\sqrt[3]{\frac{E_0}{E_1}}\right)\right].{E}_1}{0.71\sqrt[3]{\frac{E_0}{E_1}}. arctg\left(\frac{1.35\left(1.1h\sqrt[3]{\frac{E_0}{E_1}}\right)}{D}\right)+\frac{E_12}{E_op} arctg\left(\frac{D}{1.1h\sqrt[3]{\frac{E_0}{E_1}}}\right)} $$

where *h* = thickness of sub-base layer = 14 cm; *D* = design diameter = 33 cm based on 22TCN 211 (2006).

Based on Equation (2), the value *E*_1_ was 390 MPa. When using 95% of confidence level from 9 positions, the resilient modulus of steel slag was 268 MPa.

## Discussion

Table 5 shows the result of experiments conducted in this study. The steel slag can be replaced mineral aggregate applied in sub-base layer based on the requirements from TCVN 8857 and JIS A 5015.

**Table 5:** Result of experiments

No	Mechanical test	Test result	Test requirement from JIS A 5015-2018	Test requirement from TCVN 8857-2011
1	Los Angeles abrasion loss test (%)	13.9 to 17.2	-	≤35
2	CBR (%)	50.8	-	≥30
3	Immersion Expansion ratio (%)	0.31 to 0.64	≤1.0	-
4	Resilient modulus (MPa) with 95% of confidence level	268	-	-

In terms of LA abrasion test, the best mineral aggregate source in South Vietnam which locates in Tan Dong Hiep, Binh Duong province (Nguyen 2014), had about 20% bigger value than the value of steelmaking slag.

In terms of the resilient modulus, the mineral aggregate value, which had gradation of C type-based TCVN 8857, was from 150 to 200 MPa (Nguyen 2014). Then, the resilient modulus of steel slag was about 34 to 78% higher than resilient modulus of mineral aggregate. Hence, the thickness of pavement when using steel slag can be reduced about 3–5 cm in comparison with mineral aggregate in term of resilient modulus.

## Conclusion

This paper showed some preliminary results to replace steelmaking slag with the mineral aggregates in base/sub-base layer of road pavement. One gradation of steel slag was used from type C of TCVN 8857 in this study. Before creating the sample for three mechanical important test including CBR, immersion expansion, and resilient modulus test, the standard compaction test was conducted to have the optimal moisture content. The following conclusions were from the obtained results:

- The Los Angeles abrasion of steel slag was better than good mineral aggregate in South Vietnam;
- According to California Bearing Ratio and immersion expansion values, the steel slag can be used in sub-based layer of road pavement;
- The resilient modulus was very high in comparison with mineral aggregate-based natural aggregate gradation from TCVN 8857. Then, the cost of road pavement could be reduced when using steel slag for sub-base or base layer.

The obtained results also show that steel slag could be a good alternative material for mineral aggregate in application in road pavement in terms of mechanical as well as environmental properties. Moreover, this statement should be evaluated over a long time in some road sections around our country.

## Data Availability

The data presented in this study are available on request from the corresponding author. The data are not publicly available due to the information security conditions of the joint research project between JFE Steel Corporation (Japan) and Ho Chi Minh City University of Technology (Viet Nam).
